# Physical fitness of Ghanaian physiotherapists and its correlation with age and exercise engagement: a pilot study

**DOI:** 10.1186/s40945-016-0016-2

**Published:** 2016-02-24

**Authors:** Ajediran I. Bello, Emmanuel Bonney, Bridget Opoku

**Affiliations:** Department of Physiotherapy, School of Biomedical & Allied Health Sciences, College of Health Sciences, University of Ghana, Legon, Accra, Ghana

**Keywords:** Physical fitness, Exercise, Physiotherapists

## Abstract

**Background:**

The physical job demands of physiotherapists require optimal level of physical fitness (PF), which is often not evaluated in practice. In this study, we assessed selected components of physical fitness of Ghanaian physiotherapists in relation to their sex, age and frequency of exercise participation.

**Methods:**

Physiotherapists practicing in four major hospitals within the Accra Metropolis of Ghana were enrolled into this cross-sectional survey. Three major components of physical fitness (flexibility, cardiorespiratory endurance and body composition) were assessed with sit and reach test, 3-min step test and BMI respectively. Unpaired sample t-test was used to determine differences in means of the three components of physical fitness betwwen males and females. Pearson correlation coefficient showed that frequency of exercise engagement and age of the participants correlated with the three components of physical fitness at *p* < 0.05.

**Results:**

The study sample consisted of 40 participants, out of which 23 (58 %) were females. The mean age was (31.5 ± 1.4) years and majority 21 (52.5 %) was within the age range of 20-29 years. Respective mean scores for cardiorespiratory endurance, flexibility and BMI were (98.2 ± 12.9 beat/min), (4.03 ± 6.15 cm) and (23.3 ± 3.4 kg/m^2^). Female participants were significantly more flexible than their male counterparts (5.7 ± 5.3; 1.6 ± 6.6, p = 0.034). There was a positive and significant correlation between the age of the participants and BMI (r = 0.614 and p = 0.017). However, cardiorespiratory endurance was not significantly correlated with age and frequency of exercise engagement.

**Conclusions:**

The sampled physiotherapists had relatively low physical fitness compared to the age adjusted values. Age and sex are therefore crucial determinants whilst designing programmes aimed at promoting physical fitness in this group.

## Background

Delivery of high-quality and cost-effective healthcare services has increasingly become the focus of policy-makers, clinicians, and patients’ advocacy groups. To meet such high demands, health care providers including physiotherapists need to develop optimal state of health and fitness. No study has considered the fitness level of any group of health care professionals prior to the present survey in Ghana. One major existing difficulty in the estimation of physical fitness (PF) is the lack of normative data for specific population owing to varying determinants. Physical fitness remains a key requirement for good health among the general population although its assessment could be marred by various factors. Indeed, there is a close link between low PF and health outcomes of individuals [1]. In spite of the glaring consequences of the deficits in PF on health status of individuals, the World Health Organization [2] reported that, about 60 % of the global populations still do not engage in the recommended levels of physical activity to maintain optimal level of PF. For instance, the recommended physical activity level for the general population stipulates a minimum of 30-min of moderate-intensity aerobic activity for five days per week [3]. However, evaluation of PF goes beyond aerobic component only, but also encompasses muscle strength and endurance, body composition and flexibility [4, 5].

The demands of physiotherapy practice are diverse and require practitioners to demonstrate an optimal level of fitness to be able to deliver quality service to their clients and patients. The physical tasks involve safe handling and moving of patients, gait training and using body segments to provide support and resistance during treatment sessions [6]. Physiotherapists are often involved in promoting physical activity and health in both healthy subjects and individuals with medical conditions. However, fitness levels of physiotherapists have not been well explored in many developing countries like Ghana, warranting the need to investigate the fitness levels of physiotherapists working in urban areas of Ghana. Previous studies have suggested that physiotherapists are usually susceptible to work-related musculoskeletal disorders [7, 8]. Similarly, an assessment of the components of PF by Gorner et al. [9] reported that low levels of physical activity among physiotherapy undergraduate students was accompanied by lower level of aerobic capacity than that obtained in their physical education counterparts.

A population-based study on physical activity among health professionals generally showed high sedentary lifestyle [10]. This view was supported by the findings of Skaal and Pengpid [11] in which majority (81.5 %) of the sampled health workers in South Africa fell within low fitness level, 15.5 % in moderate level while only 3 % met the criteria for a high level of fitness. In the United States, although Black et al. [12] showed that physiotherapists and physiotherapy students engage in health promoting behaviours at high level, the students were found to be more likely to believe that role modeling is a powerful teaching tool for clients. In Ghana, assessment of the components of PF for students is neither stressed in the curriculum during training nor emphasized as routine criteria at the entry-level of physiotherapists’ practice. Whilst this trend raises concerns about the inherent level of PF among the practicing physiotherapists, it also emphasizes the need to consider factors that might be crucial in the attainment of optimal physical health.

In addition, low manpower in the health sector remains an unresolved issue regarding physiotherapy practice in developing countries including Ghana. It is therefore necessary to ascertain the determinants of physical health of the few available physiotherapists in order to promote good health and wellbeing. Even though a reasonable level of PF is required to execute the routine physical job demands, the common prevailing associated variables are often ignored. This study thus sought to determine the PF of Ghanaian physiotherapists with respect to their age, sex and frequency of exercise engagement.

## Methods

### Participants

Data were collected from the physiotherapy departments of Korle-bu Teaching Hospital (KBTH), 37 Military Hospital (MH), Ridge General Hospital and Tema General Hospital. The KBTH and 37 MH are tertiary health facilities located in Accra, the capital city of Ghana and attend to clients from all the ten Regions of Ghana and certain parts of West Africa. The Ridge and Tema General Hospitals are regional health facilities, located in Accra and Tema respectively. These facilities serve individuals within the Greater Accra Region of Ghana. The four health facilities have the largest concentration of physiotherapists in the southern part of Ghana numbering 48 registered members of the Ghana Physiotherapy Association.

The study involved 40 physiotherapists practicing in the above-mentioned facilities. They were recruited through sample of convenience. Participants were enrolled if they met the following inclusion criteria: Registration with the Ghana Physiotherapy Association and Allied Health Professional Council of Ghana. Participants’ were also required to have had at least one year of clinical experience. Retired physiotherapists, interns and those on short vocational visits were not included in the study.

### Materials for data collection

A 30 cm by 30 cm locally fabricated wooden box was used as a sit and reach box. The extents of excursion during sit and reach test was measured with a retractable tape measure. Body weight was measured with a digital Seca Model weighing scale to the nearest 100 g. Test re-test reliability was ensured by measuring a known weight with the scale from time to time. The height of each participant was measured to the nearest 0.1 cm with self-designed height meter calibrated from 0-200 cm (inter- rater reliability, *r* = 8.3). Timing of 3-min step test with a stopwatch determined the cardiorespiratory endurance while the sequence of the stepping was monitored with metronome. An automatic digital blood pressure monitor (USA) was used to assess the pulse rate of participants. The participants’ demographic data and the frequency of their exercise engagement were recorded in a data capturing form.

### Procedure

The protocol for this study was approved by the Ethics and Protocol Review Committee of the School of Allied Health Sciences (now School of Biomedical and Allied Health Sciences), University of Ghana. All the participants were contacted at the selected centers and the aims of the study were explained to them. Each participant gave written informed consent prior to the commencement. Information regarding sex and age of the participants, and their frequency of exercise engagement were recorded on a data capturing form by a trained research assistant. The pattern of exercise engagement per week was determined on recall by the participants. Three researchers (Physiotherapists) were involved in the physical fitness testing procedures. Each testing station was manned by a specific researcher who was assisted by a trained research assistant.

All assessments took place at each of the selected facilities at an appointed time determined in consultation with the participants. Body weight was measured in standing without shoes and in light clothing. Measurement of height was performed in standing with feet placed flat on the floor while looking straight during the measurements. The same physiotherapist measured the weight and the height of the participants. BMI was calculated using the height (meters) and weight (kilogram) respectively and the value was determined with the formula: weight (kg)/height (m) ^2^.

#### Flexibility

Participants’ flexibility was assessed through modified sit and reach test. This was performed in long sitting on exercise mats with the participants’ back at 90° and their feet against the sit and reach box. With scapular adduction as the only movement, the upper limbs were fully extended, hands superimposed and placed on the box (starting position). The tips of the middle fingers in this position mark the zero point [13]. They were asked to stretch and slide gradually forward in a straight direction on the reach box. The total excursion made was recorded as the sit and reach distance. The test was performed thrice and the average was taken to represent their flexibility.

#### Cardiovascular endurance

3-min step test was performed by the participants on 12-in. bench. They were required to step up and down the bench at a steady pace of 24 beats per minute (as pre-set on the metronome) for 3 min. Participants were asked to stop at the expiration of 3 min. The pulse rate was measured immediately after the test in sitting position.

### Data analysis

Data were analyzed using the Software Statistical Package for Social Sciences (SPSS) version 20.0. Descriptive statistics of mean and standard deviation were employed to summarize the data. Shapiro-Wilk test determined the normality of the data distribution whilst unpaired sample *t* test was used to compare the selected components of physical fitness between the male and female physiotherapists.

Pearson correlation coefficient was adopted to determine the relationship of age and frequency of exercise with the selected components of physical fitness at *p* < 0.05.

## Results

Forty (40) physiotherapists with age range 21 to 59 years (mean age = 31.5 ± 1.4 years) participated and completed the study. They were segregated into four age ranges and the majority 21 (52.5 %) were within age range 20–29 years while only 2 (5 %) were found in age range 50–59 years. 23 (58 %) of the participants were females. Eight (20 %) of the sampled physiotherapists indicated that they routinely engaged in exercise thrice weekly as compared to larger proportion 21 (52.5 %) who confirmed that they hardly engage in any specific exercise. All the participants who engaged in exercise 19(%) adopted aerobic patterns out of the which 7(%) mostly embraced jogging (Table 1).

**Table 1:** Participants’ demographics and pattern of exercise engagement

Variables	Frequency	Percentage (%)
Age		
(years)	21	52.5
30–39	12	30.0
40–49	5	12.5
50–59	2	5.0
Gender		
Male	17	58.0
Female	23	42.0
Frequency of exercise engagement		
Under exercised	21	52.5
Once	2	5
Twice	4	10
Thrice	8	20
Four times	3	7.5
Seven times	2	5
Pattern of exercise		
Cycling	3	7.5
Jogging	7	17.5
Skipping	4	10
Brisk walking	3	7.5
Group exercise	2	5

Evaluation of the data using Shapiro-Wilk test for normality of distribution showed no statistical significance for males and females as regards BMI (*p* = 0.614 and *p* = 0.425), flexibility (*p* = 0.072 and *p* = 0.063) and cardiorespiratory endurance (*p* = 0.631 and *p* = 0.326). The data showed no violation of normality for all the three components. The mean flexibility score of the participants was 4.03 ± 6.15 cm with females being more significantly flexible than their male counterparts (5.7 ± 5.3; 1.6 ± 6.6, *p* = 0.034). Overall mean scores for cardiorespiratory endurance and BMI were 98.2 ± 12.9 beats/min and 23.3 ± 3.4 kg/m^2^ respectively. There were no statistically significant differences (*p* > 0.05) in the mean scores of the male and female participants for both parameters (Table 2).

**Table 2:** Flexibility, Body Mass Index (BMI) and cardiorespiratory endurance measured in male and female participants (mean ± SD)

	Male (n = 17)	Female (n = 23)	p
BMI (kg/m^2^)	22.3 ± 2.8	24.1 ± 4.1	0.140
Cardiorespiratory endurance (Beat/min)	97.4 ± 12.1	98.7 ± 13.9	0.754
Flexibility (cm)	1.6 ± 6.6	5.7 ± 5.3	0.034

Pearson product–moment correlation was run to analyze the relationship of the participants’ age and pattern of exercise engagement with their BMI, flexibility and cardiorespiratory endurance (Table 3). Bivariate analysis indicates a strong, positive correlation between age and BMI (*r* = 0.377; *p* = 0.017). However their age has a weak, nonsignificant positive correlation with cardiorespiratory endurance (*r* = 0.038; *p* = 0.815) and a weak nonsignificant negative correlation with flexibility (*r* = −0.032; *p* = 0.846). Similarly, participants’ frequency of exercise engagement shows weak, nonsignificant positive correlation with BMI (*r* = 0.046; *p* = 0.776), but a weak, nonsignificant negative correlation with cardiorespiratory endurance (*r* = −0.182; *p* = 0.261) and flexibility (*r* = −0.083; *p* = 0.611).

**Table 3:** Association of age and frequency of exercise engagement with physical fitness measures (Pearson’s r coefficients)

	BMI	Cardiorespiratory endurance	Flexibility
Age	0.377*	0.038	−0.032
Frequency of exercise engagement	0.046	−0.182	−0.083

*p = 0.017

## Discussion

The focus of this study was to explore some intervening factors that could pre-set attainment of physical fitness among selected Ghanaian physiotherapists. Of the total physiotherapists sampled for the study, 19 (47.5) could specify their exercise engagements, which were largely aerobics. Findings from this study revealed that the participants recorded mean scores of 4.0 ± 6.2 cm, 98.2 ± 12.9beats/min and 23.3 ± 3.7 kg/m^2^ for flexibility, cardio-respiratory endurance and body mass index respectively. Although the overall mean BMI was within the normal range stipulated by WHO, the obtained scores for the flexibility and cardiorespiratory endurance were below the average for age-adjusted values [14]. This finding is not surprising as larger proportion 21 (52.5 %) of the sampled physiotherapists seem to fall short of any healthy physical fitness exercise despite their expected knowledge of its benefits.

This finding corroborates the outcome of a study in Brazil on the same topic in which sedentary lifestyle was found to be frequent among health professionals [10]. It is therefore suggestive that physiotherapists are no exception. Many factors could be implicated as root causes of poor exercise engagement among the physiotherapist including job schedule and work demands of individuals. The two factors sufficed in Ghana given the high ratio of physiotherapist to patients in the health care system. Although the ratio varies from country to country, a study in Australia revealed a ratio of 1 physiotherapist to 12 clients in 2008 [15] while it was 1: 100,000 in Ghana in the same year [16]. Most physiotherapists are therefore not able to add routine physical exercises to their busy schedules. These factors might have contributed to the outcome of the present study.

Female participants scored significantly higher than male participants on the flexibility test. A study by Ortega et al., [17] also reported a similar finding where females performed better in sit and reach test (flexibility) than their male peers.

Spernoga et al., [18] and Depino et al., [19], adduced higher values of flexibility in females to difference in viscoelastic properties of muscles on gender basis. Again, of all the selected components, participants’ BMI was found to be significantly and positively correlated with their age, which implies increase in adiposity in older age. This finding follows similar trend with the previous study [9]. Logically, it could be argued that there is likelihood of decline in exercise engagement and physical activities with increasing age occasioned by intrinsic physiological factors, which could ultimately lead to slow rate of fat decomposition.

The weak negative statistical relationships between the frequency of exercise engagement and cardiorespiratory endurance implies that fewer exercise engagement resulted in high pulse rate (low cardiorespiratory endurance). This finding explains the poor state of exercise engagement among the participants in which only about 13 (32.5 %) followed the recommended frequency of exercise performance per week i.e. exercising for 30 min at least thrice per week. Although pulse rate and arteriovenous oxygen difference are useful measures for estimating oxygen consumption, pulse rate can be increased by sympathetic drive under a variety of circumstances other than aerobic training [20]. Thus, the high pulse rate recorded in this study in spite of its linear relationship with oxygen consumption cannot be used as surrogate for aerobic capacity of the participants. More so, duration for pulse rate recovery following exercise varies from within 5 min to one hour depending on the exercise intensity and modality used [21]. Hence, ours finding on participants’ response based on pulse rate should be taken with caution.

Given that the health-promoting behaviour that stands to receive the greatest attention from physiotherapists is the promotion of physical activity among patients, it demands of them to be physically active, not only for their personal health benefits, but also to allow them to present themselves as better role models [22]. The current recommendations for physical activity advocated by the American College of Sports Medicine and the American Heart Association stipulate that healthy adults aged 18 to 65 years need to engage in moderate-intensity physical activity for a minimum of 30 min, 5 days each week or vigorous-intensity physical activity for a minimum of 20 min, 3 days each week [3]. The sampled participants in this study fall short of this trade off which puts demand for adequate sensitization by relevantl regulatory body.

### Limitation

Although this study has unveiled the fitness status of a cross section of physiotherapists in Ghana, the sample size is still too small. This forms the major limitation in this study as a sizeable proportion of the sampled population declined to partake in this study. Also, inclusion of other components such as muscular strength and muscular endurance would have provided more information with regard to their fitness level. Again, the use of cycle ergometry would have allowed a more in-depth assessment of endurance as against the 3-min step test. Within the limitation of this study nonetheless, our findings have provided an insight into the health status of the physiotherapists in Ghana.

## Conclusion

In conclusion, the significant impact of sex and age in relation to flexibility and BMI suggests their importance as useful determinants to be considered to encourage the attainment of physical fitness among physiotherapists, particularly cardiorespiratory endurance and flexibility. These findings have created an opportunity for the Ghana Physiotherapy Association to entreat her members about routine exercise participation. Future study is being planned to consider larger sample size to evaluate all the components of physical fitness among the Ghanaian Physiotherapists.
