# Erratum to: Physical fitness of Ghanaian physiotherapists and its correlation with age and exercise engagement: a pilot study

**DOI:** 10.1186/s40945-016-0017-1

**Published:** 2016-03-21

**Authors:** Ajediran I. Bello, Emmanuel Bonney, Bridget Opoku

**Affiliations:** Department of Physiotherapy, School of Biomedical & Allied Health Sciences, College of Health Sciences, University of Ghana, Legon, Accra Ghana

## Erratum

After publication of this work [1], it was noted that we inadvertently failed to include the complete list of co-authors. This has been corrected here, and in the original article. We apologize for any inconvenience this oversight may have caused.
